# The Urine Circulating Cathodic Antigen (CCA) Dipstick: A Valid Substitute for Microscopy for Mapping and Point-Of-Care Diagnosis of Intestinal Schistosomiasis

**DOI:** 10.1371/journal.pntd.0002008

**Published:** 2013-01-24

**Authors:** José Carlos Sousa-Figueiredo, Martha Betson, Narcis B. Kabatereine, J. Russell Stothard

**Affiliations:** 1 Disease Control Strategy Group, Liverpool School of Tropical Medicine, Liverpool, United Kingdom; 2 Department of Infectious and Tropical Diseases, London School of Hygiene and Tropical Medicine, London, United Kingdom; 3 Vector Control Division, Ministry of Health, Kampala, Uganda; Centers for Disease Control and Prevention, United States of America

## Abstract

**Background:**

The World Health Organization now recommends the provision of praziquantel treatment to preschool-aged children infected with schistosomiasis. For intestinal schistosomiasis the current operational field diagnostic standard is examination of a thick Kato-Katz smear by microscopy prepared from a single stool specimen, and although pragmatic, this methodology has well-known shortcomings. Here, as a potential alternative, the performance of the urine circulating cathodic antigen (CCA) dipstick test was assessed in terms of disease-mapping and point-of-care diagnosis for intestinal schistosomiasis in preschool-aged children. Our manuscript reports on findings at baseline and at the end of a one-year longitudinal treatment study.

**Methodology/Principal Findings:**

A total of 925 children (mean age 2.8 years) were initially recruited from six lakeshore villages representative of high, moderate and low levels of disease transmission. At baseline, all children were tested for intestinal schistosomiasis by microscopic examination of duplicate Kato-Katz smears prepared from a single stool faecal, by antigen detection with the urine CCA dipstick test and by serology with a commercially available ELISA test (as ‘gold-standard’) that measures host antibody titres to soluble egg antigens. As a point-of-care diagnosis, the urine CCA dipstick test achieved sensitivity and specificity values ranging from 52.5–63.2% and 57.7–75.6%, respectively, with faecal microscopy achieving very high specificities (>87%) but sensitivities as low as 16.7% in the low transmission setting.

**Conclusion/Significance:**

The urine CCA test was shown to be more effective than faecal microscopy especially in lower transmission settings. The diagnostic performance of this test was not significantly impacted by treatment history or co-infections with other intestinal helminths.

## Author Summary

The World Health Organization (WHO) now recommends that all children (2–16 year olds) living in schistosomiasis endemic areas should have regular access to praziquantel treatment. While there is extensive information in current literature on the geographical distribution of this disease in school-aged children (6–16 year olds), very little is known about its distribution in infants and preschool-aged children. Although pragmatic for resource-poor settings, the current field-standard method, the Kato-Katz smear, lacks diagnostic sensitivity, especially in lower transmission settings or where there is a large proportion of recently acquired infections. The latter is of particular importance for preschool-aged children as many will have pre-egg patent infections. Here we investigate a commercial rapid diagnostic test alternative to microscopy, using a dipstick test that detects worm antigens in the patient's urine, and show that it can achieve better performance than the current field standard both during mapping efforts and as a point-of-care diagnosis. Importantly, we found this diagnostic tool to be as effective before and after praziquantel treatment, able to provide semi-quantitative information on intensity of infection, and to be far more sensitive for identifying recently acquired infections than any other present alternatives.

## Introduction

Preschool-aged children (<6 year olds) from sub-Saharan Africa are now recognised as living at risk from both urogential (caused by *Schistosoma haematobium*) and intestinal (caused by *S. mansoni*) schistosomiasis, and several studies have shown that treatment with praziquantel (PZQ) is both safe and efficacious [1]–[4]. Consequently, the World Health Organisation (WHO) is now recommending that young children living in schistosome-endemic areas should be considered for treatment with PZQ during child health campaigns at the standard dose of 40 mg/kg [5]. The importance of identifying cases in early childhood is two-fold: firstly, to provide treatment with PZQ and arrest development of morbidity from active infections acquired early in childhood, and secondly, tackling infections in this younger age-group may well have an added effect in reducing local environmental contamination and putative schistosome transmission [6].

Current WHO guidelines for mapping and routine surveillance of intestinal schistosomiasis rely upon detecting eggs after microscopic examination of a single Kato-Katz thick smear prepared from a single stool specimen [7]–[8]. Although the Kato-Katz technique can also provide quantitative information on egg intensity and act as a proxy for host worm burden, to perform detailed egg-counts is often considered unnecessary during mapping initiatives or in point-of-care (POC) settings when only a qualitative result (i.e. presence of eggs) is required. By undertaking a qualitative examination, the microscopist is able to provide a quicker turnaround time for each examined smear. The sensitivity of a single examination, however, can be very low due to a combination of well-known factors, such as the variation in the distribution of eggs within a single stool specimen and day-to-day variation in faecal egg concentrations especially when host egg excretion is sporadic [9]–[12]. Additionally, in this young age class where infections are likely recently acquired (i.e. pre-egg patent) or of low intensity, these could also remain largely undetected by direct egg detection methods such as microscopy owing to a temporal lag in diagnostic patency as worms mature to fecundity [13].

According to recent reviews on helminth research, appropriate and, more often than not, distinct diagnostic methodologies are required for: 1) disease mapping to guide initiation and prioritisation of control interventions; 2) monitoring and evaluation of ongoing control, and being particularly alert to the detection of possible emerging anthelmintic resistance; 3) assessment of elimination of disease by intervention programmes as these approach completion; and 4) case-based diagnosis for surveillance and patient management [14]–[15]. Currently, faecal microscopy is the field standard for 1) and 4) in much of sub-Saharan Africa.

Therefore the objective of this study was to ascertain if the diagnostic performance of the commercially available urine Circulating Cathodic Antigen (CCA) dipstick test was comparable to that of faecal microscopy (i.e. duplicate thick Kato-Katz smears from a single stool) for disease mapping and POC diagnosis in preschool-aged children. The CCA rapid diagnostic test (Rapid Medical Diagnostics, Pretoria, RSA) is an immunochromatographic dipstick that detects the presence schistosome antigens (proteoglycans), as released from feeding worms, in host urine [16]–[17]. Prior applications of this test in school-aged children have yielded diagnostic sensitivities of between 56.3–96.3% and specificities as high as 93.9% [18]–[23]. Initial observations from surveys of preschool-aged children indicate similar values, albeit with lower specificity [13], [24]. In the search of a diagnostic ‘gold standard’, our study also considered host serology, by assessing antibodies titres to schistosome soluble egg antigen (SEA), as being most sensitive, though this methodology is typically too demanding for wide-scale application in field-based settings in sub-Saharan Africa.

This study focused on the detection of *S. mansoni* infection in preschool-aged children (≤6 year olds) at baseline and at one-year follow-up during a PZQ treatment study campaign that took place in Uganda in 2009–2011. The longitudinal performance of the CCA dipstick test was compared to faecal microscopy, as well as host serological dynamics. The logistical and financial characteristics of each test were also discussed here to make a pragmatic assessment of the urine CCA dipstick within a context of disease mapping and POC diagnosis.

## Materials and Methods

### Ethical statement, recruitment and treatment

The London School of Hygiene and Tropical Medicine, London, UK (application no. LSHTM 5538.09) and the Ugandan National Council of Science and Technology approved this study. Before selection, all families received an information leaflet (in local languages) detailing the objectives and procedures of this study. Those who chose to participate had the study explained in full by the local Vector Control Disease District Officer. Before enrolment, informed consent was given by mothers in writing or by fingerprint (in cases of illiteracy). The IRB approved the use of oral consent.

At baseline, all children and their mothers (guardians) were treated using a standard 40 mg/kg dose of PZQ (CIPLA, Mumbai, India) regardless of infection status in line with mass drug administration guidelines. Treatment at 3- and 6-month follow-ups was provided on a selective basis upon positive criterion of either faecal examination or CCA test in an effort to understand (re)infection dynamics within the cohort. In addition albendazole (ALB) (GSK, Uxbridge, UK), was provided following WHO deworming guidelines [25]. All treatment was supervised and confirmed by a project nurse. For younger/smaller children (<24 months old), PZQ tablets were broken and crushed, mixed with a spoonful of orange juice, before administration. All children were provided with additional juice and a food item (local doughnut) at the time of treatment to ensure treatment was not taken on an empty stomach and in an attempt to improve absorption of the drug.

### Study site

This is a retrospective study, where the baseline data was collected in October/November of 2009 in Bugoigo, Walukuba and Piida villages (Buliisa District) on the shores of Lake Albert and Bugoto, Bukoba and Piida villages (Mayuge District) on the shores of Lake Victoria. The follow-up study took place in October/November of 2010. The sites visited are characterized as fishing/lakeshore villages impoverished both in terms of sanitation and hygiene, and have been the setting for the Schistosomiasis in Mothers and Infants (SIMI) project [6], [26].

### Inclusion and exclusion criteria for analysis

**Figure 1:**
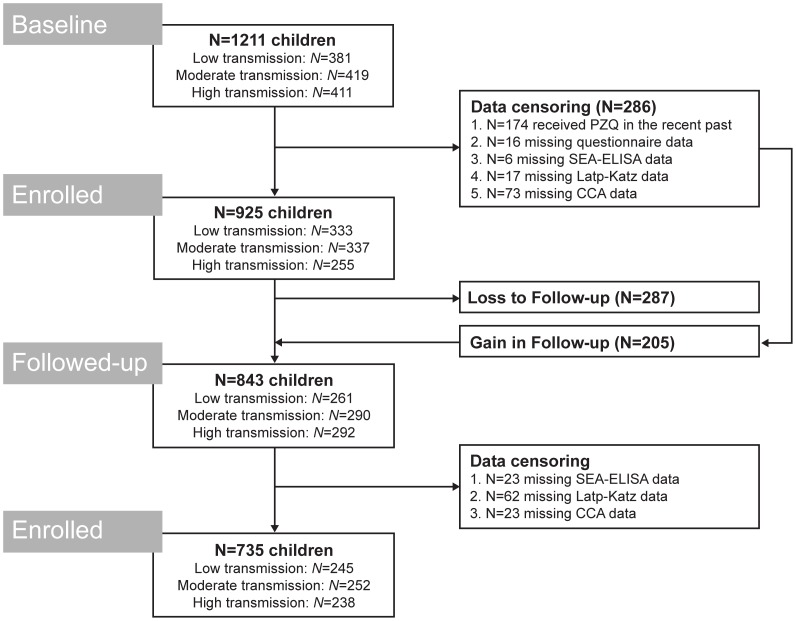
Flowchart detailing the study participation and adherence to sample submissions for diagnosis of *S. mansoni*. According to single Kato-Katz thick smear examinations, the prevalence of *S. mansoni* in preschool children living in the high, moderate and low transmission settings was 7.2%, 16.9% and 38.8%, respectively. Study was conducted in October/November 2009 and 2010.

In order to be included in this study, all participants had to meet the following criteria at baseline: 1) aged below 6 years at recruitment; 2) had no history of previous treatment for schistosomiasis; and 3) provided sera, urine and stool samples. All children who had been recruited into the study at baseline (N = 1211) were considered for follow-up as long as criterion 3 (above) was met. Importantly, upon follow-up 69% of children had received at least two doses of PZQ in the previous 12 months, one at baseline and a second at an interim 3- or 6-month survey. The remaining 31% of children received only one treatment (at baseline). For details on population numbers achieved, see Figure 1.

### Parasitological diagnosis

Note that the five technicians in charge of diagnostic tests were blinded to results achieved by other methodologies.

#### Microscopy

Diagnosis of *S. mansoni* was performed using duplicate Kato-Katz thick smears prepared from a single stool sample (41.7 mg of stool per smear) [7]. Microscopy was conducted by three experienced Ministry of Health technicians and supervised by a senior technician for quality control. Results were expressed as eggs per gram of faeces (epg) and infection intensities of *S. mansoni* were categorised as follows: 1–99 epg as light, 100–399 epg as medium and ≥400 epg as heavy infections according to WHO guidelines [25]. Thick smears were also examined for presence of infections by hookworm, *Ascaris lumbricoides* and *Trichuris trichiura* (the common soil-transmitted helminths, STH), as well as *Hymenolepis nana* (dwarf tapeworm) and *Enterobius vermicularis* (pinworm). Infections with *Strongyloides stercoralis* in these settings are negligible [27].

#### Urine Circulating Cathodic Antigen (CCA) dipstick test

A single urine sample from each child provided a 50 µl aliquot for testing the presence of schistosome CCA with a commercially available immuno-chromatographic dipstick (Rapid Medical Diagnostics, Pretoria, RSA), a rapid diagnostic test for intestinal schistosomiasis [28]. This test was performed by a trained Ministry of Health technician with extensive experience with rapid diagnostic tests. To facilitate better recording of the visual intensity of the CCA reaction band within the test zone, results were visually graded against a reference chart for: trace, single (+), double (++) and triple (+++) positive reactions. Trace reactions were considered positive.

#### Serology

A commercially available ELISA kit (IVD Inc.; Carlsbad, USA) was used to test for host antibodies (IgM & IgG) to schistosome soluble egg antigens (SEA) according to manufacturer's instructions. This test was performed by a Liverpool School of Tropical Medicine researcher with extensive experience in laboratory techniques. Briefly, approximately 75 µl of finger-prick blood was taken from each child, clotted and sera was harvested by pipette after centrifugationthen diluted 1∶40 with specimen dilution buffer before loading a total of 100 µl into each ELISA microwell. ELISA reactions were read using a portable ELISA plate reader (Labtech International Ltd., East Sussex, UK) to quantify optical density (OD) at 450 nm. Samples with over 0.2 OD/450 nm were considered positive; a further classification was employed whereby samples with OD at 450 nm between 0.2 and 2.0 and those between 2.1 and 3 were considered positive (+) and strong positive (++). The dynamics of antibody titres to SEA in these children (and mothers) were recorded across transmission settings with regard to new infections (i.e. seroconversions), as well as changes after PZQ treatment.

### Performance as prevalence estimation tools

Prevalence values estimated based from results of faecal microscopy, the urine CCA test and SEA-ELISA were compared to prevalence values if all techniques were combined (‘combined result’) and expected values if triple sample microscopy had been carried out using a predictive mathematical model [29].

### The CCA dipstick test as a POC diagnostic

Faecal microscopy and the urine CCA test were tested qualitatively as alternative intestinal schistosomiasis diagnosis methodologies against the SEA-ELISA. Typically serology serves as the ‘gold-standard’ for diagnosis of travellers, young children living in endemic settings or individuals from low-transmission settings although it can be confounded by history of PZQ treatment [30]. We also used a second ‘gold standard’ by considering a positive test result (regardless of the test) as true-positive, as before [31]. Hence, we combined results from all tests (i.e., duplicate Kato-Katz thick smears from single stool sample, single urine CCA test and single SEA-ELISA) and therefore maximized specificity. For each, sensitivity and specificity were calculated at baseline and after treatment.

Receiver operating characteristics (ROC) analyses were also performed [32], plotting the true positive rate (Sensitivity) in function of the false positive rate (100-Specificity). Calculation of standard error (SE) of area under the ROC curve was performed according to [33]. For ROC analysis, the SEA-ELISA was considered the ‘gold-standard’ (as a binomial variable) against which results from faecal microscopy (as a binomial variable) and the urine CCA test (as a continuous variable whereby: −ve = 0, trace = 1, + = 2, ++ = 3, +++ = 4) were measured for performance.

### Statistical analysis

Statistical analysis was carried out according for each of the three different endemic settings separately. Villages were classified according to results from faecal microscopy: prevalence level <10% (‘low transmission’), 10–25% (‘moderate transmission’) and >25% (‘high transmission’).

Data were collected from each individual using pre-format data sheets, which were then entered onto an electronic format using Microsoft Excel. The data thus collated were analysed using MS Excel and R statistical package v 2.10.1 [34]. For prevalence values and measures of diagnostic performance, 95% confidence intervals (CI_95_) were estimated using the exact method as described in [35]. Percentage comparisons were performed using (one-tailed) Fisher's exact modification of the 2×2 chi-squared test, with *P*-values inferior to 0.05 deemed significant [36]. For infection intensity values, the geometric mean of Williams, GM_W_ [37] was chosen as the measure of central tendency due to the typical overdispersion present in this type of data, and (asymmetric) CI_95_ values were estimated according to [38]. Data were analysed and presented as in similar publications by authors from the Schistosomiasis Consortium for Operational Research and Evaluation (SCORE) in an attempt to standardize data reporting relating to diagnostic performance of the CCA test [20], [23].

## Results

### Study population

At baseline, of 925 preschool-children were allocated into the study (female to male ratio = 0.91, mean age = 2.8 years), 94 infants (<12 months of age), 130 one year olds, 183 two year olds, 171 three year olds, 180 four year olds, 165 five year olds and 2 six year olds. Upon follow-up (Figure 1), 735 of the initial 925 preschool-children were recovered (female to male ratio = 0.91, mean age = 4.0 years), 53 one year olds, 101 two year olds, 138 three year olds, 143 four year olds, 152 five year olds and 148 six year olds. There were no significant differences in sex ratio and ages between the three transmission sites.

The majority of the children were born in the village surveyed (81%) and had access to pit latrines (92%), most of which were used by the immediate family only (i.e. non-communal, 88%). At baseline, only 22 children were reported as to having ever urinated blood (9 girls and 13 boys), with unknown aetiology, and no macro-haematuria was observed in any of the children's urine samples. A much larger proportion, however, were reported to have passed blood in stool in the recent past (38%). A total of 12% of children reported knowing how to swim; 69% of children reported spending less than 30 min per day in the lake water while 24% reported spending between 30 min and one hour and 7% reported spending more than one hour daily.

### Prevalence of *S. mansoni* infection

**Figure 2:**
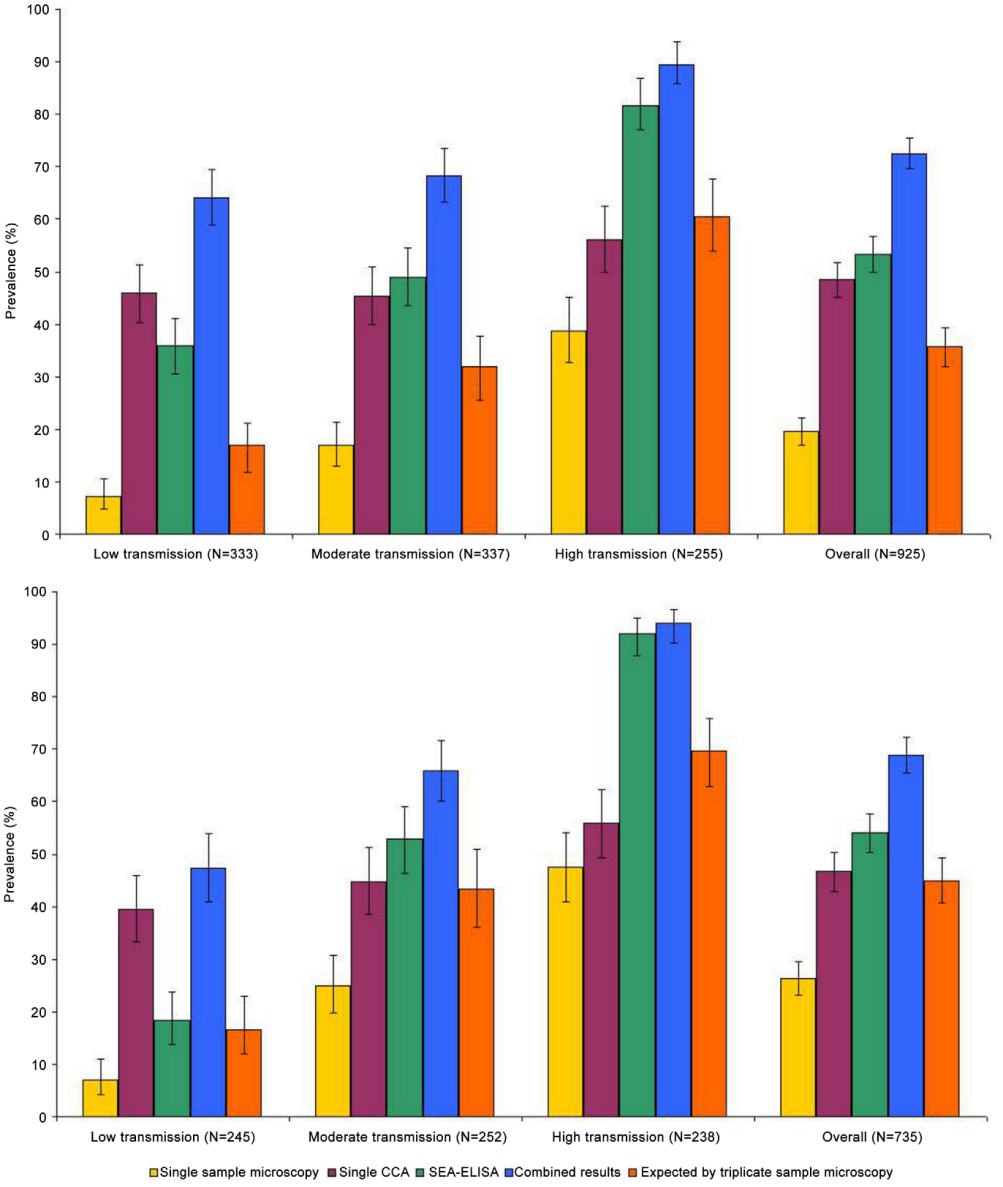
Prevalence of intestinal schistosomiasis in preschoolers at baseline and follow-up. Microscopy was conducted on duplicate Kato-Katz thick smears from the same stool sample; a single CCA tests was conducted on a single urine sample; SEA-ELISAs were conducted in the field; ‘combined results’: if positive for any test then considered child as positive; and mathematical model by Jordan and colleagues (‘Triplicate stool samples microscopy’) [29] Baseline results represented in top section, and follow-up results represented in bottom section.

The prevalence of *S. mansoni* egg-patent infections according to faecal microscopy was 40.3% (CI_95_ 31.8–49.3%) in Bugoigo, 16.9% (CI_95_ 12.3–22.5%) in Bugoto, 6.9% (CI_95_ 3.9–11.1%) in Bukoba, 7.8% (CI_95_ 3.6–14.3 in Lwanika, 16.8% (10.4–25.0%) in Piida and 37.3% (28.9–46.4%) in Walukuba (Table 1 and Figure 2). Consequently, villages were grouped as follows according to endemicity: Bukoba and Lwanika were considered of ‘low transmission’, Bugoto and Piida were considered of ‘moderate transmission’ and Bugoigo and Walukuba were considered of ‘high transmission’ (see Figure 1).

**Table 1:** Prevalence of intestinal schistosomiasis in preschool-aged children.

Diagnostic approach	Low transmission		Moderate transmission		High transmission	
	Baseline	Follow-up	Baseline	Follow-up	Baseline	Follow-up
*N*	333	245	337	252	255	238
Faecal microscopy	7.2 (4.7–10.5)	6.9 (4.1–10.9)	16.9 (13.0–21.4)	25.0 (19.8–30.8)	38.8 (32.8–45.1)	47.5 (41.0–54.0)
Light infections (1–99 epg)	6.3 (4.0–9.5)	6.5 (3.8–10.4)	10.4 (7.3–14.1)	17.0 (12.6–22.3)	24.3 (19.2–30.1)	27.3 (21.8–33.4)
Moderate infections (100–399 epg)	0.9 (0.2–2.6)	0.4 (0.0–2.3)	4.7 (2.7–7.6)	4.8 (2.5–8.2)	7.8 (4.9–11.8)	9.3 (5.9–13.7)
Heavy infections (≥400 epg)	0.0 (0.0–1.1)	0.0 (0.0–1.5)	1.8 (0.7–3.8)	3.2 (1.4–6.2)	6.7 (3.9–10.5)	10.9 (7.3–15.6)
Single urine CCA	45.9 (40.5–51.5)	39.6 (33.4–46.0)	45.4 (40.0–50.9)	44.8 (38.6–51.2)	56.1 (49.8–62.3)	55.9 (49.3–62.3)
trace reaction	27.9 (23.2–33.1)	26.1 (20.7–32.1)	25.8 (21.2–30.8)	24.6 (19.4–30.4)	26.3 (21.0–32.1)	16.8 (12.3–22.2)
+ reaction	13.8 (10.3–18.0)	11.0 (7.4–15.6)	13.1 (9.7–17.1)	15.1 (10.9–20.1)	14.5 (10.4–19.4)	17.2 (12.7–22.6)
++ reaction	3.6 (1.9–6.2)	1.6 (0.4–4.1)	2.4 (1.0–4.6)	3.6 (1.6–6.7)	5.1 (2.7–8.6)	11.8 (8.0–16.6)
+++ reaction	0.6 (0.0–2.2)	0.8 (0.0–2.9)	4.2 (2.3–6.9)	1.6 (0.4–4.0)	10.2 (6.8–14.6)	10.1 (6.6–14.6)
SEA-ELISA	36.0 (30.9–41.4)	18.4 (13.7–23.8)	49.0 (43.5–54.4)	52.8 (46.4–59.1)	81.6 (76.3–86.1)	92.0 (87.8–95.1)
+ reaction	32.7 (27.7–38.1)	18.4 (13.7–23.8)	40.1 (34.8–45.5)	48.8 (42.4–55.2)	61.6 (55.3–67.6)	78.2 (72.4–83.2)
++ reaction	3.3 (1.7–5.8)	0.0 (0.0–1.5)	8.9 (6.1–12.5)	4.0 (1.9–7.2)	20.0 (15.3–25.4)	13.9 (9.7–18.9)
Combined tests	64.0 (58.6–69.1)	47.3 (40.9–53.8)	68.2 (63.0–73.2)	65.9 (60.0–71.7)	89.4 (85.0–92.9)	94.1 (90.3–96.7)
‘Triplicate sample microscopy’	17.0 (12.9–22.3)	16.6 (11.9–22.9)	32.0 (26.2–38.4)	43.4 (36.1–50.9)	60.5 (53.4–67.2)	69.7 (62.9–75.9)

Prevalence (and CI_95_), in %, of intestinal schistosomiasis according to the different diagnostic tests employed (faecal microscopy, single urine CCA and SEA-ELISA), the combined results for all tests (if positive for any test then considered child as positive), as well as the mathematical model by Jordan and colleagues (‘Triplicate stool samples microscopy’) [29]. This study was conducted in three epidemiological settings in Uganda in October/November 2009 (baseline) and 2010 (follow-up). For each stool sample, duplicate Kato-Katz thick smears were performed.

#### Faecal microscopy

In low, moderate and high transmission settings, the observed prevalence of egg-patent schistosomiasis according to faecal microscopy was 7.2%, 16.9% and 38.8% before treatment and 6.9%, 25.0% and 47.5% one year after treatment (*P*>0.05). At baseline, the GM_w_ of infection intensities identified by faecal microscopy were 0.29 epg (CI_95_ 0.00–1.35 epg, max. value 336 epg) in the low transmission setting, 1.01 epg (CI_95_ 0.00–2.16, max. value 2820 epg) in the moderate transmission setting, and 4.61 epg (CI_95_ 1.33–5.88 epg, max. value 4140 epg), with prevalence of heavy infections reaching 1.8% and 6.7% in the moderate and high transmission settings, respectively. Upon follow-up, the GM_w_ of infection intensities identified by faecal microscopy were 0.26 epg (CI_95_ 0.00–1.33 epg, max. value 336 epg) in the low transmission setting, 1.71 epg (CI_95_ 0.00–2.92, max. value 1536 epg) in the moderate transmission setting, and 7.98 epg (CI_95_ 4.67–9.29 epg, max. value 2556 epg), with prevalence of heavy infections reaching 3.2% and 10.9% in the moderate and high transmission settings, respectively.

#### CCA test results

In low, moderate and high transmission settings, the observed prevalence *S. mansoni* infections according to CCA dipsticks was 45.9%, 45.4% and 56.1% before treatment and 39.6%, 44.8% and 55.9% one year after treatment (*P*>0.05), respectively. Most of the positive diagnoses were single positive reactions, with few double and triple positive reaction recorded. For prevalence (and CI_95_) of each reaction intensity see Table 1.

#### SEA-ELISA test results

In low, moderate and high transmission settings, the observed prevalence *S. mansoni* infections according to SEA-ELISA was 36.0%, 49.0% and 81.6% before treatment and 18.4% (P<0.001 compared to baseline), 52.8% (*P*>0.63 compared to baseline) and 92.0% (P<0.001 compared to baseline) one year after treatment, respectively. Most of the positive reactions were classified as positive. For prevalence (and CI_95_) of each reaction intensity see Table 1.

### Prevalence of other infections

Other helminths were identified by faecal microscopy at baseline: 2.1% (CI_95_ 1.3–3.3%) of children were positive for *T. trichiura*, 10.1% (CI_95_ 8.2–12.3%) were positive for hookworm infections, 1.7% (CI_95_ 1.0–2.8%) were positive for *H. nana* and 0.3% (CI_95_ 0.0–0.9%) were positive for *E. vermicularis*. No *A. lumbricoides* was identified. As for follow-up, prevalence of other helminths according to faecal microscopy was as follows: 0.1% (CI_95_ 0.0–0.8%) of children were positive for *A. lumbricoides*, 1.1% (CI_95_ 0.5–2.1%) were positive for *T. trichiura*, 4.6% (CI_95_ 3.2–6.4%) were positive for hookworm infections, 1.4% (CI_95_ 0.7–2.5%) were positive for *H. nana* and 0.5% (CI_95_ 0.1–1.4%) were positive for *E. vermicularis*.

### Diagnostic accuracy for POC

#### Baseline

When considering the SEA-ELISA the ‘gold standard’, the overall sensitivity for faecal microscopy was 33.1% (CI_95_ 28.9–37.4%) and for the urine CCA test was 56.4 (CI_95_ 51.9–60.8%). The overall specificity for faecal microscopy was 96.1% (CI_95_ 93.8–97.7%) and for the urine CCA test was 60.4% (CI_95_ 55.6–65.1%). When considering the combined results variable as ‘gold standard’, the overall sensitivity of faecal microscopy was 26.8% (CI_95_ 23.5–30.3%), for the urine CCA test was 66.9% (CI_95_ 63.2–70.5%), and for the SEA-ELISA was 73.5% (CI_95_ 70.0–76.8%). For details on diagnostic performance in each of the prevalence settings refer to Table 2.

**Table 2:** Diagnostic performance of different diagnostic tests for *S. mansoni* infections before and after a treatment.

	Low transmission		Moderate transmission		High transmission	
	Sensitivity, % (CI_95_)	Specificity, % (CI_95_)	Sensitivity, % (CI_95_)	Specificity, % (CI_95_)	Sensitivity, % (CI_95_)	Specificity, % (CI_95_)
**Before treatment**						
**SEA-ELISA as ‘gold-standard’**						
Faecal microscopy	16.7	98.1	30.3	95.9	44.7	87.2
	(10.5–24.6)	(95.3–99.5)	(23.4–37.9)	(91.8–98.3)	(37.8–51.7)	(74.3–95.2)
Single CCA	52.5	57.7	55.2	64.0	59.6	59.6
	(43.2–61.7)	(50.8–64.5)	(47.2–62.9)	(56.3–71.1)	(52.6–66.3)	(44.3–73.6)
**Combined results as ‘gold-standard’**						
Faecal microscopy	11.3	100.0	24.8	100.0	43.4	100.0
	(7.4–16.3)	(97.0–100.0)	(19.3–30.9)	(96.6–100.0)	(36.9–50.1)	(87.2–100.0)
Single CCA	71.8	100.0	66.5	100.0	62.7	100.0
	(65.3–77.8)	(97.0–100.0)	(60.0–72.6)	(96.6–100.0)	(56.1–69.0)	(87.2–100.0)
SEA-ELISA	56.3	100.0	71.7	100.0	91.2	100.0
	(49.4–63.1)	(97.0–100.0)	(65.4–77.5)	(96.6–100.0)	(86.8–94.6)	(87.2–100.0)
**After treatment**						
**SEA-ELISA as ‘gold-standard’**						
Faecal microscopy	20.0	96.0	37.6	89.1	50.7	89.5
	(9.6–34.6)	(92.3–98.3)	(29.3–46.4)	(82.0–94.1)	(43.9–57.5)	(66.9–98.7)
Single CCA	60.0	65.0	63.2	75.6	58.4	73.7
	(44.3–74.3)	(58.0–71.6)	(54.4–71.4)	(66.9–83.0)	(51.6–65.0)	(48.8–90.9)
**Combined results as ‘gold-standard’**						
Faecal microscopy	14.7	100.0	38.0	100.0	50.4	100.0
	(8.8–22.4)	(97.2–100.0)	(30.5–45.8)	(95.8–100.0)	(43.7–57.2)	(76.8–100.0)
Single CCA	83.6	100.0	68.1	100.0	59.4	100.0
	(75.6–89.8)	(97.2–100.0)	(60.4–75.1)	(95.8–100.0)	(52.6–65.9)	(76.8–100.0)
SEA-ELISA	38.8	100.0	80.1	100.0	97.8	100.0
	(29.9–48.3)	(97.2–100.0)	(73.2–85.9)	(95.8–100.0)	(94.9–99.3)	(76.8–100.0)

This study was conducted in three epidemiological settings in Uganda in October/November 2009 (baseline) and 2010 (follow-up). For each stool sample, duplicate Kato-Katz thick smears were performed.

#### Follow-up

When considering the SEA-ELISA the ‘gold standard’, the overall sensitivity for faecal microscopy was 42.8% (CI_95_ 37.8–47.9%) and for the urine CCA test was 60.2% (CI_95_ 55.2–65.1%). The overall specificity for faecal microscopy was 93.2% (CI_95_ 90.0–95.6%) and for the urine CCA test was 69.2% (CI_95_ 64.0–74.1%). When considering the combined results variable as ‘gold standard’, the overall sensitivity of faecal microscopy was 38.1% (CI_95_ 33.9–42.5%), for the urine CCA test was 67.8% (CI_95_ 63.5–71.8%), and for the SEA-ELISA was 78.5% (CI_95_ 74.6–82.0%). For details on diagnostic performance in each of the prevalence settings refer to Table 2.

### ROC analysis

**Figure 3:**
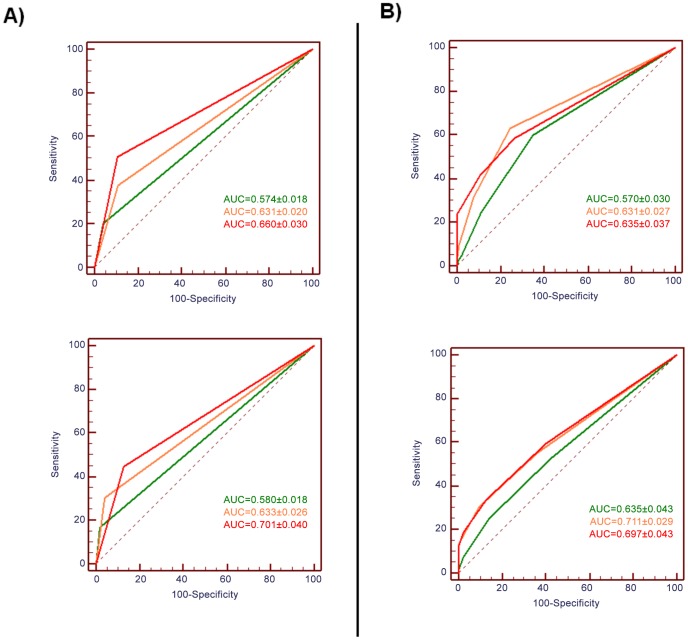
ROC curves of microscopy and the CCA using SEA-ELISA results as reference test. The receiver operating characteristic (ROC) curves and the area under the curve (AUC) of faecal microscopy (A) and CCA (B) are presented from either a low (green), moderate (orange) or high transmission (red) setting. Results are presented from baseline (above) and follow-up (below) surveys.

The ROC curves and AUC of microscopy and the urine CCA according to setting are presented in Figure 3. It appears that the discriminating powers of single CCA was higher as transmission increased, as the values of AUC were 0.57, 0.63 and 0.64 in the low, moderate and high transmission setting, respectively. The same was observed for microscopy, with AUC values of 0.57, 0.63 and 0.66 in the low, moderate and high transmission setting, respectively. A similar dynamic was observed at follow-up for both tests with AUC values increasing, albeit not significantly. Of note was the observation that considering urine CCA trace results as negative results rendered the test highly specific in all transmission environments, before and after treatment (specificity >85%). As a consequence, however, sensitivity dropped (SS<45%), particularly in low and moderate transmission environments (SS<30%).

## Discussion

Preventive chemotherapy campaigns typically rely on disease mapping exercises to identify and prioritise areas where infections are a public health issue to maximize long-term cost-effectiveness [14]. This type of rapid identification of high risk communities warranting treatment can also serve as a platform for further research related to descriptions of host morbidity, PZQ drug performance, monitoring and evaluation, as well as improved patient management [39]. With the known shortcomings of the current field diagnostic standard, i.e. microscopic examination of a single stool sample [9]–[12], there is ample reason to develop other methods that attempt to reveal the ‘true’ prevalence of infection, thus alternative, field-applicable methods are needed. To this end, use of rapid diagnostic tests is appropriate. This is of particular importance in the case of detecting early infections in very young children, as the health burden in this age-class is being more formally explored and these children are now being recommended for treatment during health campaigns [5]. Here our analyses has attempted to shed new light on the diagnostic performance of faecal microscopy (duplicate KK smear) and a commercially available rapid diagnostic test, the urine CCA dipstick, in three different disease transmission settings in Uganda, with recourse to host serological dynamics. It is worth of note that *S. haematobium* is not endemic in these regions of Uganda, so cryptic urogenital schistosomiasis is unlikely to perturb subsequent diagnostic findings [40].

### SEA-ELISA as the ‘gold-standard’?

As with all diagnostic studies with schistosomiasis, the issue of a ‘gold standard’ (or even a ‘silver’ diagnostic standard) is contentious given the patient history and stage of infection to be assessed, i.e. acute or chronic. Identifying the ‘best’ standard was crucial during the concept stages of this study. While triplicate stool sampling may have been an appropriate methodology for detecting infection in school-aged children, in young children many infections are at a pre-egg patent stage or produce very low faecal egg counts so this method is flawed even at a conceptual level. In *S. mansoni* infections, egg production usually begins at the end of adult maturation with at least 30–50 days from skin penetration to egg-laying [41], and even then, most low faecal egg counts will still remain undetected by microscopy as egg fail to pass into the intestinal lumen [9]–[12]. Due to the shortfalls of microscopy, the authors opted for the more expensive, more sensitive, albeit less field-applicable, ELISA as the ‘gold standard’. Antibody-based assays are well-recognised as being very sensitive [42]–[43] but cannot always distinguish between an active or past infection, which is important in the context of regular provision of PZQ to active infections. However, they can be extremely useful for infection incidence studies in children and in low-transmission settings [30]. Antibody responses to egg antigens are commonly detectable four to six weeks after contact with contaminated water [44], rendering ELISA-based diagnosis more sensitive than microscopy, especially at these early stages. The combined dynamics of IgM and IgG responses to schistosome infection in young children in the endemic setting, however, are as yet only beginning to be fully determined, especially when also set against the background of PZQ treatment(s).

The fact that the SEA-ELISA was used as the ‘gold-standard’ after all children received at least one round of treatment in the 12 months preceding the follow-up survey must also be discussed. Humoral responses have been known to remain in patients treated for schistosomiasis for up to 24 months [45]. However, results from the SIMI project in Uganda indicate that while some individuals remained antibody-positive for the duration of the study, many were in fact still egg-positive for infection despite treatment. On the other hand, a significant proportion of individuals (both mothers and children) seroconverted to negative within a six and twelve month window after initial treatment (see Figure S1). As these titres reflect the combined titre levels of IgM and IgG, which may be considered a shortcoming of this commercial kit, there might be some short- and long-term trends manifesting themselves concurrently. In future a more precise discrimination between these antibody sub-classes, especially IgG sub-types, would be beneficial. Additionally, the results presented here show that the correlation between egg excretion, presence of CCA antigens in urine and presence of antibody responses against egg antigens remains very similar before and after treatment (See Figure S2). Therefore we consider the SEA-ELISA a viable alternative ‘gold standard’ (compared to microscopy) for detecting infection in very young children even after treatment, a particularly novel finding.

### The urine CCA test in mapping initiatives

The results reported here showed that urine CCA tests gave higher prevalence levels of *S. mansoni* than single and estimated triplicate sample microscopy, the latter being calculated using a previously validated mathematical model [29], particularly in low and moderate transmission settings. Interestingly, in low transmission environments, at both baseline and follow-up, the urine CCA test identified more infections than the SEA-ELISA test, here used as the ‘gold-standard’. This is possibly due to the latency between active infection (metabolising worms), as detected by the urine CCA, and commencement of egg production and antibodies to thereof as detected by the microscopy and ELISA, respectively. The temporal relationship between eggs, antigens and antibodies is complex and has been discussed elsewhere in greater detail [13].

### The quantitative properties of the CCA dipstick

In this study we found that prevalence of CCA and ELISA strong reactions (+++ and ++, respectively) was strongly correlated to prevalence of heavy intensity infections (≥400 epg) in all settings, even after PZQ treatment. This highlights the quantitative properties of the commercial CCA test and provides evidence of its immediate applicability in mapping, monitoring and evaluation exercises. The strong association between colour intensity of the urine CCA test reaction and egg excretion values identified by microscopy is in line with previous reports (see Figure S2) [18]–[20], [46]–[47] illustrative of strong co-associations, leading the authors to conclude that a single urine CCA test provides quantitative information, along with qualitative information as reliable as that gathered by microscopic examination of a single stool sample [47]–[48].

### The urine CCA test in a POC setting

The sensitivity of single CCA varied between 53–63% when compared to the SEA-ELISA, with specificity varying between 58–76%. Contrarily, the sensitivity of faecal microscopy varied between 17–20% in the low transmission setting and 45–51% in the high transmission setting, with specificities remaining always above 87%. Importantly, ROC analysis showed that if trace results were not considered as positive diagnosis by the urine CCA test, sensitivity would vary between 24–42% and specificity would remain above 85% throughout the study. This means that in a POC scenario, considering CCA trace results as negative would render the test as sensitive and specific as single sample (double smear) microscopy. The diagnostic performance reported here falls within previously published intervals, despite the fact that our study focused on very young children and differed in the ‘gold standard’ applied [13], [18]–[24].

Noteworthy are the individuals with egg-patent infection according to microscopy but negative for CCA at baseline (47 of the 180 egg-patent, 39 light, 4 moderate and 4 heavy infections). A lower proportion was found upon follow-up (36 of 193 egg-patent, 35 light and 1 moderate infection). While the latter phenomenon may be explained by the assumption that some children still expel eggs previously trapped in the tissues long after their original infecting adult worm population was cleared by treatment, the first phenomenon was raised previously [23] and warrants further investigation to allow for optimization of diagnostic performance. Interestingly, at both time points, a vast proportion of these infections were diagnosed via observation of a single egg across both smears (19 of 57 at baseline and 19 of 35 upon follow-up). Also of note was the fact that a similar phenomenon, albeit smaller scale, was observed for SEA-ELISA, whereby 17 egg-patent children at baseline (10 light, 4 moderate and 3 heavy infections) and 22 egg-patent children upon follow-up (20 light, 1 moderate and 1 heavy) had a negative antibody titre against *Schistosoma* eggs. This phenomenon could be explained by immune incompetence. Importantly, human error must also be considered if trying to understand these observations; families could have mixed samples, technicians could have mislabelled upon sample retrieval or results could have been incorrectly entered.

### Financial and logistical implications

When considering any diagnostic test in the setting of large-scale control interventions, a variety of new hurdles arise which may make the test unattractive even if the diagnostic performance is proven satisfactory. Perhaps the foremost advantage of the Kato-Katz method is that it allows for diagnosis of other, commonly co-endemic, helminth infections, such as the STH. Furthermore, if applied to its full potential, it can also provide a quantitative measure to the infections; an aspect crucial during monitoring an evaluation campaigns where reductions in overall infection intensities are considered. Nonetheless while the CCA cannot have any bearing on diagnosis of STH, we show that it is able to act as a proxy of infection intensities.

The test's affordability is often another major constraint, and at first glance the price tag on each CCA test (approximately US$2) could be a deterrent, but a recent publication shows that to achieve similar sensitivity, especially in low and moderate transmission environments, triplicate sample microscopy will be even more expensive [49]. Importantly, the price of the CCA dipstick could still drop if the test is endorsed by the international community as a viable alternative to single sample microscopy (increased demand). As the company currently manufacturing the CCA dipstick is based in Africa it is well-placed to address the needs across the continent, aiding in reduced shipping costs.

Noteworthy is the fact that the urine CCA is a much faster methodology, which for specimen collection and in terms of mapping initiatives leads to cost-savings. Additionally, it uses a far more attainable biological sample than microscopy, with most pre- or school-aged children being capable of promptly producing a urine sample. These issues have been discussed elsewhere [20], [23]–[24], [50]. According to the literature, co-endemicity of *S. haematobium* [20] and other soil-transmitted helminths [22]–[23] does not appear to influence the accuracy of the urine CCA test for the diagnosis of *S. mansoni*.

### Conclusion

The CCA is a viable alternative for diagnosis of *S. mansoni* infections in preschool-aged children, particularly in low transmission settings or in areas where treatment has reduced prevalence to low levels. The urine CCA commercial test was proven more sensitive than faecal microscopy (duplicate KK smear) for mapping proposes, and it was as reliable as faecal microscopy for point-of-care diagnosis. The urine CCA dipstick test is able to provide both qualitative and quantitative information of *S. mansoni* infection in pre-school aged children. Importantly, these statements hold true even in the presence of regular PZQ treatment.

## Supporting Information

Figure S1 **SEA-ELISA optical density (OD, at 450 nm) dynamics in the SIMI cohort (children and mothers).** SEA-ELISAs were conducted in the field; data are reported according to transmission setting: A and D are high transmission settings, B and E are moderate transmission settings and C and F are low transmission settings. In red are individuals that had a very strong positive ELISA reaction by the end of the study (one year), in yellow are those with strong ELISA reactions by the end of the study, and in green are individuals negative for antibodies against *Schistosoma* spp. eggs by the end of the study.
(TIF)

Figure S2 **Overall correlation between microscopy, urine CCA and SEA-ELISA results at baseline (left) and follow-up (right).** Microscopy was conducted on duplicate Kato-Katz thick smears from the same stool sample; a single CCA tests was conducted per urine sample; SEA-ELISAs were conducted in the field. Urine CCA test bands were classified visually (baseline: 476 negatives, 374 +ves, 33 ++ves; and 42 +++ves; follow-up: 392 negatives, 272 +ves, 41 ++ves; and 30 +++ves); SEA-ELISA reaction strength was classified by spectrophotometer (baseline: 432 negatives, 401 +ves and 92 ++ves; follow-up: 338 negatives, 354 +ves and 43 ++ves).
(TIF)

Checklist S1 **STARD checklist.**
(DOCX)
