# Supplementary figures and images for: The Urine Circulating Cathodic Antigen (CCA) Dipstick: A Valid Substitute for Microscopy for Mapping and Point-Of-Care Diagnosis of Intestinal Schistosomiasis
Source: PLoS Negl Trop Dis. 2013 Jan 24;7(1):e2008. doi: 10.1371/journal.pntd.0002008 (PMC3554525; doi:10.1371/journal.pntd.0002008)

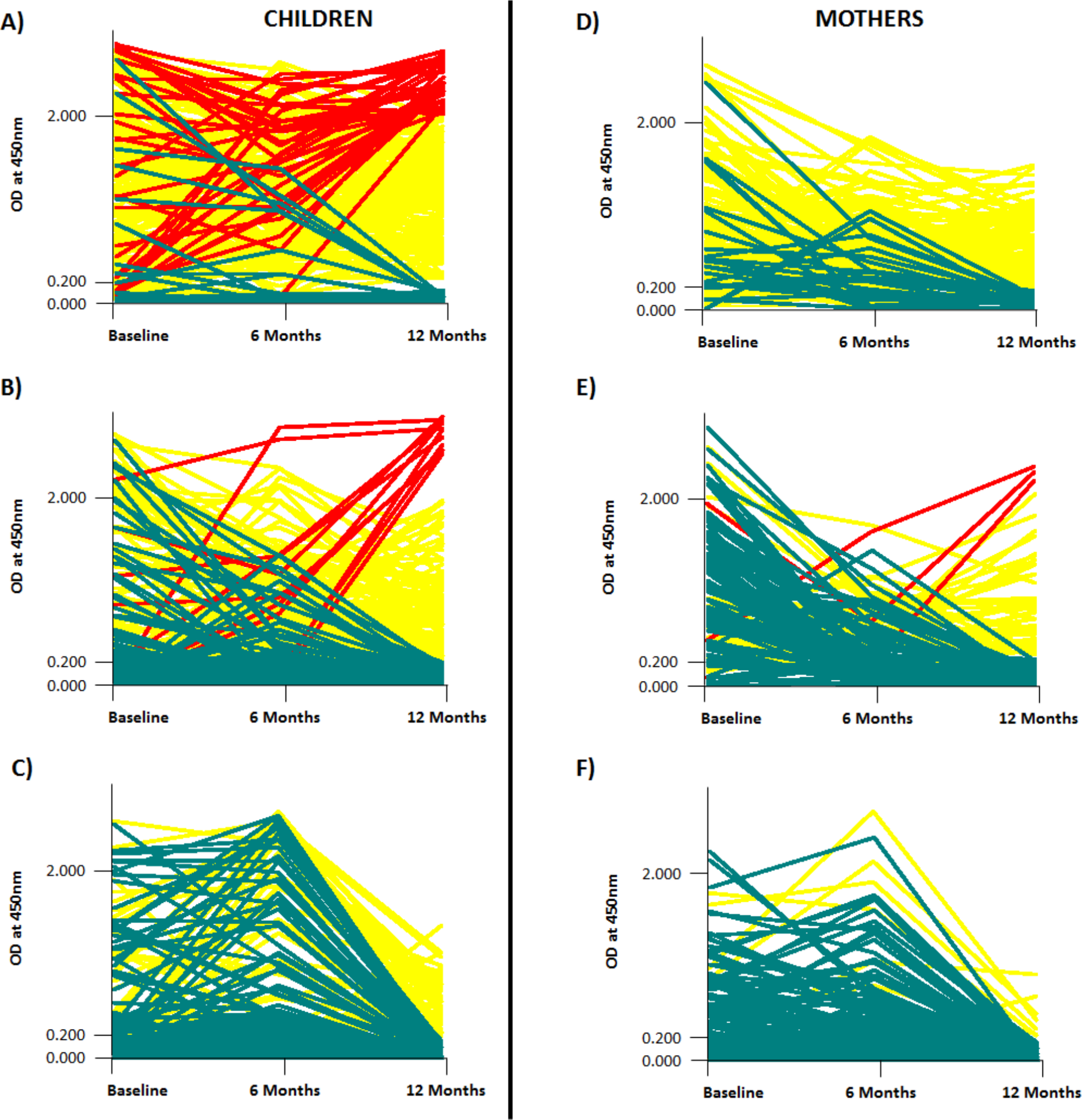

Supplement: Figure S1 — SEA-ELISA optical density (OD, at 450 nm) dynamics in the SIMI cohort (children and mothers). SEA-ELISAs were conducted in the field; data are reported according to transmission setting: A and D are high transmission settings, B and E are moderate transmission settings and C and F are low transmission settings. In red are individuals that had a very strong positive ELISA reaction by the end of the study (one year), in yellow are those with strong ELISA reactions by the end of the study, and in green are individuals negative for antibodies against Schistosoma spp. eggs by the end of the study. (TIF) [file pntd.0002008.s001.tif]

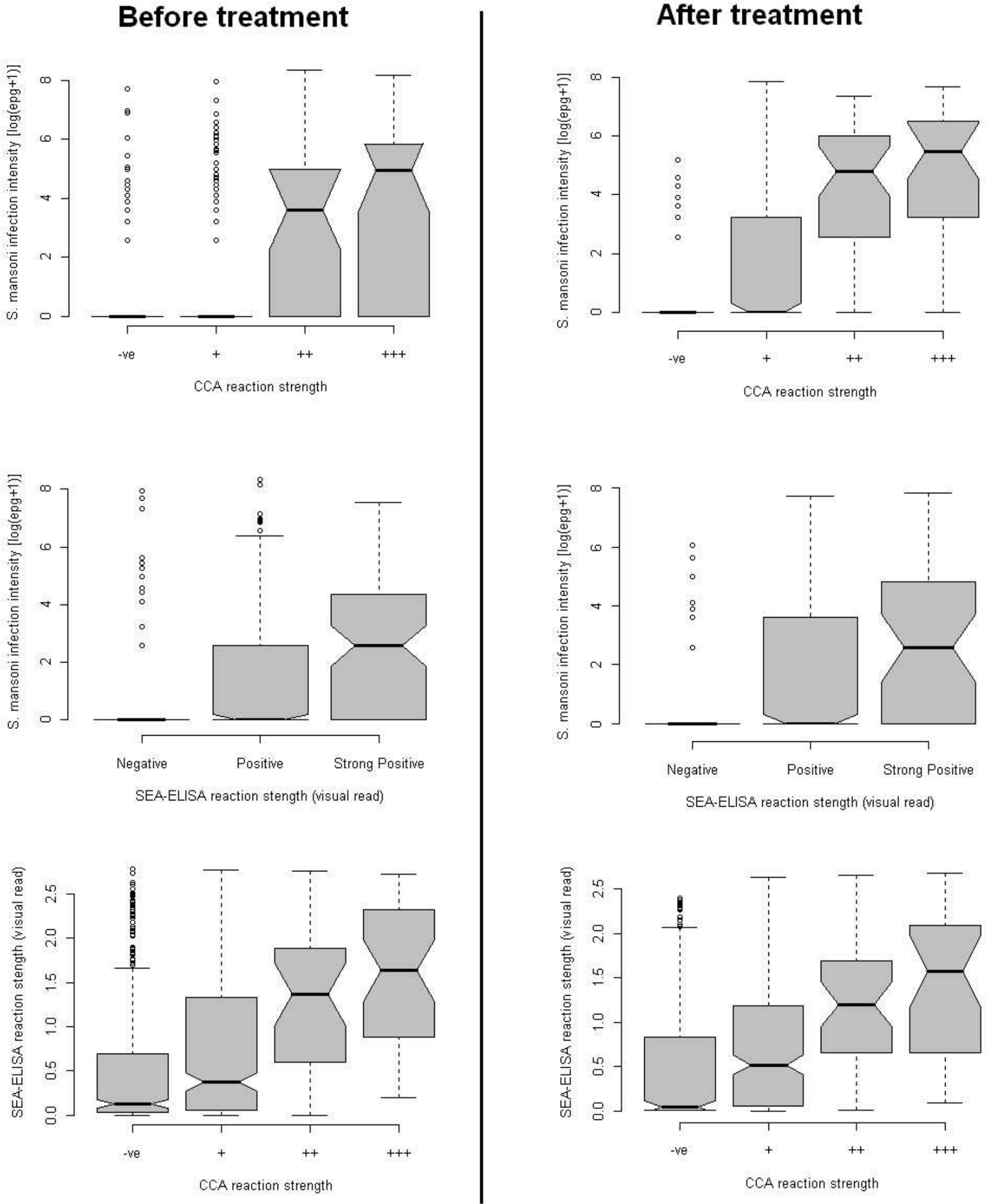

Supplement: Figure S2 — Overall correlation between microscopy, urine CCA and SEA-ELISA results at baseline (left) and follow-up (right). Microscopy was conducted on duplicate Kato-Katz thick smears from the same stool sample; a single CCA tests was conducted per urine sample; SEA-ELISAs were conducted in the field. Urine CCA test bands were classified visually (baseline: 476 negatives, 374 +ves, 33 ++ves; and 42 +++ves; follow-up: 392 negatives, 272 +ves, 41 ++ves; and 30 +++ves); SEA-ELISA reaction strength was classified by spectrophotometer (baseline: 432 negatives, 401 +ves and 92 ++ves; follow-up: 338 negatives, 354 +ves and 43 ++ves). (TIF) [file pntd.0002008.s002.tif]
